# Chronic Wound Management: From Gauze to Homologous Cellular Matrix

**DOI:** 10.3390/biomedicines11092457

**Published:** 2023-09-04

**Authors:** Valentin Popescu, Victor Cauni, Marius Septimiu Petrutescu, Maria Madalina Rustin, Raluca Bocai, Cristina Rachila Turculet, Horia Doran, Traian Patrascu, Angela Madalina Lazar, Dragos Cretoiu, Valentin Nicolae Varlas, Bogdan Mastalier

**Affiliations:** 1 General Surgery Clinic, Colentina Clinical Hospital, 020125 Bucharest, Romania; popescu.vali.umf@gmail.com (V.P.); mariuspetrutescu@yahoo.co.uk (M.S.P.); angelalazar.2008@yahoo.com (A.M.L.); bogdanmastalier@yahoo.com (B.M.); 2 General Surgery Department, Carol Davila University of Medicine and Pharmacy, 8 Eroii Sanitari Blvd., 050474 Bucharest, Romania; mariamadalinarustin@gmail.com (M.M.R.); raluca.bocai@gmail.com (R.B.); cristina_turculet22@yahoo.com (C.R.T.); doranh2003@yahoo.com (H.D.); patrascutraian@gmail.com (T.P.); 3 Urology Clinic, Colentina Clinical Hospital, 020125 Bucharest, Romania; victorcauni@yahoo.com; 4 Prof. I. Juvara General Surgery Clinic, Dr. I. Cantacuzino Clinical Hospital, 011437 Bucharest, Romania; 5 Fetal Medicine Excellence Research Center, Alessandrescu-Rusescu National Institute for Mother and Child Health, 020395 Bucharest, Romania; 6 Department of Genetics, Carol Davila University of Medicine and Pharmacy, 8 Eroii Sanitari Blvd., 050474 Bucharest, Romania; 7 Department of Obstetrics and Gynaecology, Filantropia Clinical Hospital, 011171 Bucharest, Romania; 8 Department of Obstetrics and Gynaecology, Carol Davila University of Medicine and Pharmacy, 37 Dionisie Lupu St., 020021 Bucharest, Romania

**Keywords:** chronic wounds, regenerative medicine, complex wounds

## Abstract

Background: Chronic wounds are a significant health problem with devastating consequences for patients’ physical, social, and mental health, increasing healthcare systems’ costs. Their prolonged healing times, economic burden, diminished quality of life, increased infection risk, and impact on patients’ mobility and functionality make them a major concern for healthcare professionals. Purpose: This review offers a multi-perspective analysis of the medical literature focusing on chronic wound management. Methods used: We evaluated 48 articles from the last 21 years registered in the MEDLINE and Global Health databases. The articles included in our study had a minimum of 20 citations, patients > 18 years old, and focused on chronic, complex, and hard-to-heal wounds. Extracted data were summarized into a narrative synthesis using the same health-related quality of life instrument. Results: We evaluated the efficacy of existing wound care therapies from classical methods to modern concepts, and wound care products to regenerative medicine that uses a patient’s pluripotent stem cells and growth factors. Regenerative medicine and stem cell therapies, biologic dressings and scaffolds, negative pressure wound therapy (NPWT), electrical stimulation, topical growth factors and cytokines, hyperbaric oxygen therapy (HBOT), advanced wound dressings, artificial intelligence (AI), and digital wound management are all part of the new arsenal of wound healing. Conclusion: Periodic medical evaluation and proper use of modern wound care therapies, including the use of plasma-derived products [such as platelet-rich plasma (PRP) and platelet-rich fibrin (PRF)] combined with proper systemic support (adequate protein levels, blood sugar, vitamins involved in tissue regeneration, etc.) are the key to a faster wound healing, and, with the help of AI, can reach the fastest healing rate possible.

## 1. Introduction

Due to high costs, chronic wounds have become a substantial burden to healthcare systems. The latest data on the subject have shown that the global prevalence of chronic wounds lies at 1.67 per 1000 population [1]. Most chronic wounds refer to chronic leg ulcers, and their computed prevalence is 1.51 per 1000 [2]. Of these, the most reported ethology is venous disease. Women are more affected than men, and the mean age of the chronic wound population is around 70–80 years [3]. The numbers can be even higher because of many underreported cases and a dearth of epidemiology data from low-income countries. The data on decubitus pressure injuries, which are the second most frequent, showed highly variable prevalence rates across regions, fluctuating from 0.168 cases per 1000 population in Central American females to 2.324 cases per 1000 population in North African and Middle Eastern females [1]. A study from the UK in 2016 shows that, in Europe, 1.5–2 million people are affected by acute or chronic wounds [4]. Wounds are managed in hospitals, community care centers, or at home, making the evaluation of the medical evolution and costs much more difficult. Globally, diabetic ulcers have a prevalence of 6.3%, and in Europe, this is 5.1%. The higher prevalence is in males and patients with type 2 diabetes [5].

**Figure 1:**
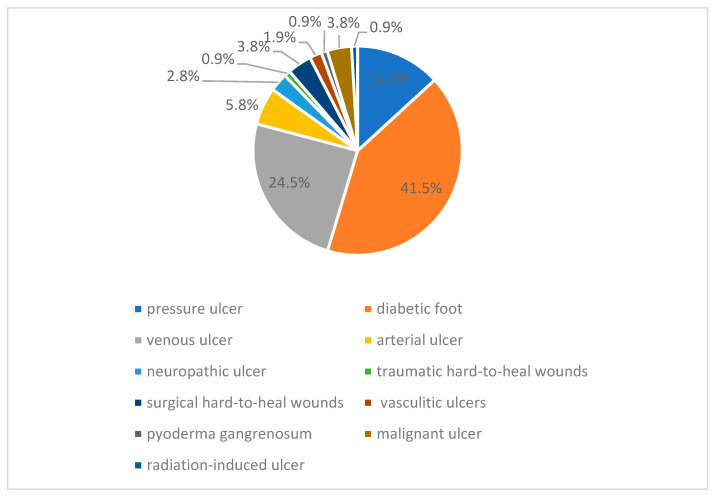
Local distribution of chronic wounds from 1-year perspective in Colentina Clinical Hospital.

Studies demonstrated that diabetic foot ulcer occurrence has a significant and independent impact on lower extremity amputation and death, suggesting that prevention is the key to increasing life quality and decreasing mortality [6]. Diabetic foot ulcers result from a combination of factors, including neuropathy (nerve damage), peripheral vascular disease (poor blood circulation), and impaired wound healing, often triggered by diabetes-related metabolic abnormalities, which collectively contribute to reduced sensation, increased pressure, and compromised tissue integrity in the feet, leading to ulceration and difficulty in wound healing. The annual incidence rate of amputation was 109.63 per 100,000 people with diabetes in the US. Furthermore, the annual mortality rate in patients with diabetic foot ulcers is about 11% and about 22% for those with an incident lower extremity amputation (5,6) [7,8]. As presented in Figure 1, more than half of the chronic wounds affect the foot area and are composed of diabetic foot ulcers, venous ulcers and arterial ulcers.

Initial evaluation of the wound management costs indicates that wound dressings (which are materials used such as dressings, bandages, or topical antiseptics) might seem to be the significant cost factor. However, nursing time and hospital care costs account for approximately 80–85% of the total cost [4]. Therefore, the materials and treatments chosen for each case can significantly influence the total cost. Most of the costs include inpatient services (professional fees, staffing costs, and equipment costs), pharmaceuticals, and other expenses directly related to health care delivery. The time necessary for wounds to heal, the frequency of dressing changes, and medical complications are the major factors for cost rises [9]. Therefore, modern and advanced technology targeting more rapid wound healing can substantially reduce the management cost. The “hard-to-heal” lesions, which are called chronic wounds, are defined as lesions that fail to heal with “standard therapy” in the medical predicted time and parameters [7]. As a result, they cause further deterioration in health and quality of life, and increase the burden on the healthcare system over a long period. 

Scientific data collected between 2000 and 2015 indicate that the cost burden was mainly attributed to amputation costs for patients with associated diseases such as diabetes. For example, in the articles reviewed, the hospitalization cost in the United States of America ranged between USD 12,851 and USD 16,267 [8]. In Europe, diabetic foot ulcer management reaches EUR 4–6 billion per year [10].

Uncomplicated surgical wounds are not exceedingly expensive to manage, but costs increase substantially if wound infection occurs. For example, a European study shows that surgical wound infection adds, on average, 11 days of hospitalization, with an average cost of EUR 5800 per case [11]. For the data evaluated from the USA, the cost of treating a surgical site infection (SSI) adds around USD 20,000 to the bill [12]. USA data show that venous ulcers cause a loss of 2 million working days/year, and as a result, the US healthcare system spends around USD 2.5–3.5 billion annually [13].

A retrospective study from 2016 in the UK that followed a cohort over one year estimated that the cost per healed wound ranged from EUR 805€ to EUR 4611 per patient. The cost of an unhealed wound ranged from EUR 1982 to EUR 6892 per patient, making it 135% more than that of a healed wound [14].

A study in the UK from 2009 estimated that SSIs add 11 days per episode (on average) to the total hospitalization period of a patient with chronic wounds [10]. A heavy infection of chronic wounds is a major cause of non-healing and has a significant role in increasing the cost of healthcare [15]. Around 13.3% of leg ulcers and 10.4% of pressure ulcers became infected during treatment. One of the consequences of wound infections for non-healing chronic wounds is receiving systemic antibiotics, and over 60% of the patients received at least one antibiotic treatment over six months. In addition, the occurrence of osteomyelitis brings an additional cost of treatment for Category III and IV pressure ulcers, estimated to be more than EUR 34,564 in the UK. This cost includes biopsies, MRIs, antibiotics, microbiological tests, and surgery [4].

The primary burden that wound healing has on the medical economy can be solved by strategies that focus on wound prevention, accurate diagnosis, and improving wound healing rates, which could generate clinical and economic benefits for both patients and healthcare systems.

Patients with chronic wounds have a poor quality of life (from a medical perspective) and wound-related costs are substantial. Thus, the need to develop and implement a chronic wound management strategy that focuses on increasing health-related quality of life and effectively reducing costs for this patient group is urgently needed.

The main objective of this review is to provide healthcare specialists (physicians, nurses, and other wound care providers) with a complex view of the problem of chronic wounds and to give them information on how to personalize the treatment for each case for a better outcome. At first, the focus is on recognizing the wound stage and finding the optimal stage of therapy. Then, the focus goes to evaluating the options that better suit the case.

## 2. Materials and Methods

We evaluated 48 articles from years 2000 to 2022 registered in the MEDLINE and Global Health databases. The articles included in our study had a minimum of 20 citations, included patients >18 years old, and focused on chronic, complex, hard-to-heal wounds. Searches were performed manually, using the PubMed Advanced Search Builder (https://pubmed.ncbi.nlm.nih.gov/advanced/, accessed on 17 June 2023), searching the “Title/Abstract” section for the following key terms, combined: “chronic wound management”, “modern chronic wound therapy”, “PRP therapy chronic wound”, “PRF therapy chronic wound”, “NWPT chronic wound”, “alternative therapy chronic wounds”, “AI chronic wound” and “chronic wound protection and treatment”.

Extracted data were summarized into a narrative synthesis and used the same health-related quality of life impact. The central objective of this study was to observe how the concept of chronic wound care has evolved, from the materials used to the pathophysiological concepts, in order to improve the current care protocols. Although each type of chronic wound (diabetic ulcer, venous ulcer, pressure injuries, etc.) has its therapeutic peculiarities, the emphasis of the work falls on the common elements.

## 3. Wound Healing

**Figure 2:**
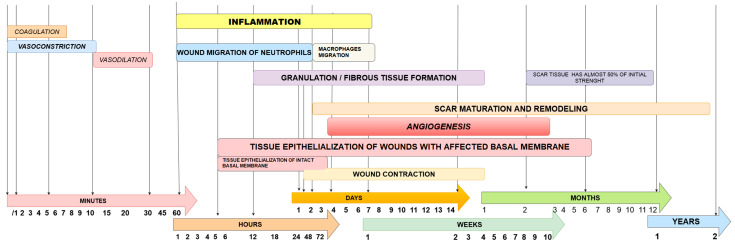
Stages of tissue healing. The first hour after the tissue damage is regulated by vascular reactions and, almost every time, passes to the next phase, in which the main event is the inflammation where most of the wounds get stuck and, after 4 days, the main event that can hold back a wound from healing is the formation of new blood vessels.

Wound healing is a physiological process achieved through four organized, chronologically, and spatially overlapping phases that begin immediately after the injury, as seen in Figure 2. Each one has its specific purpose and cellular/molecular factors, which allow, all together, the contiguous minimization of internal damages, the cellular cleansing of the damaged area, and the needed structural repair. Wound healing is achieved through hemostasis, inflammation, proliferation, and remodeling of tissues [16]. Since 1950, several terminologies have been used to define the term “chronic wound” to encompass a common pathophysiological aspect and a sense of duration. A widely accepted definition is “wounds that have not proceeded through an orderly and timely reparation to produce anatomic and functional integrity after three months” [17]. In the literature, other descriptions can be found, characterizing the lesion as a barrier defect that has not healed in 3 months [18], or as any wound lacking a 20–40% reduction in size after 2 to 4 weeks of optimal treatment, or when there is no evidence of complete healing after 6 weeks [19]. Even though chronic wounds may differ from an etiological point of view, certain standard features can be seen. For instance, there are consistently excessive levels of pro-inflammatory cytokines, proteases, and reactive oxygen species (ROS), a lack of growth factor (GF) and extracellular matrix (ECM) secretion, as well as dysfunctional and insufficient number of stem cells [16].

As soon as the lesion occurs, platelets from the injured vessels are activated to promote clot formation and stop the hemorrhage. After clotting, platelets release factors to attract the immune cells from the blood vessels and into the wound, making the first stage of the inflammatory phase. Polymorphonuclear neutrophils are the first responders, followed by monocytes that quickly differentiate into macrophages [20]. Neutrophils are responsible for high levels of reactive oxygen species (ROS), proteolytic enzymes, and pro-inflammatory cytokines, all secreted to clean the wound. After the successful debridement of the wound, neutrophils enter apoptosis, being phagocytized by the macrophages [20].

In order to enter the proliferative phase, the wound needs to be sterilized, and necrotic tissue has to be removed. In the proliferative stage of healing, immature cells from the wound margins and wound bed proliferate and migrate to regenerate the lost tissue. The primary cell types participating in wound repair are endothelial cells, fibroblasts, and keratinocytes. At first, fibroblasts migrate to the extracellular matrix (ECM) and form the granulation tissue, and keratinocytes cover the surface to close the wound [20]. Remodeling, the final phase of wound healing, aims to increase the mechanical strength of scar tissue [21]. The final stage of healing is to obtain neovascular tissue, ending with a collagen-rich scar [22].

### 3.1. Wound Assessement

When assessing a wound, multiple parameters should be considered for classification—location, size, edges, type of tissue in the base, depth, and surrounding skin [23]. Size measurement is not very accurate but provides an efficient and easy way to assess the progression of wound closure over time. It should be carried out at the first presentation and regularly after that. Depth is another parameter that can be used to determine wound progression but is less practical than the aforementioned external method. A detailed assessment of the wound edges can help the examiner to determine its etiology. In the case of any suspicious wound, a biopsy should be taken. The type of tissue present at the base of the wound provides valuable information relative to the expectation of total healing time and the risk of complications, including the infectious status of the wound. The wound bed may cover three types of non-viable tissue, namely, necrotic tissue, slough, or eschar, which also help the medical team to choose an appropriate debridement type to manage the wound [23], as seen in Figure 2.

An adequate wound bed appreciation and preparation must be the first step in wound healing therapy [24] and repeated as much as needed because, at this stage, medical professionals can guide the optimal therapy [25]. From 2015 to 2020, video technology evolved and generated mobile software applications for wound assessment. Currently, there are powerful wound assessment tools available, such as +WoundDesk (D+Wound Solution) and Mobile Wound Care (Tissue Analytics), which can evaluate the wound healing progression with more and more accuracy. Mobile and tablet software now allows healthcare providers to document any wound with accuracy and precision, some of them having a less than 5% error rate on wound measurements, with fully automatic wound/lesion measurements, while offering real-time data insights for evidence-based research [26]. All of the data collected by these mobile applications are processed into an extensive database. The applications connect medical professionals with their patients with a continuous real-time assessment of the wound/s. Over time, hospitals, clinics, and patients save time and money with the wound data collected and analyzed online.

Additional parameters can detect the presence of an active or underlying infection. These parameters are evaluating the base of the wound, the surrounding skin, the odor, and the color of the wound—as well as the level of pain the patient feels [16,23]. Wound infections are associated with heavy exudates from the wound bed and need a radical change in wound management, including appropriate antimicrobial dressings. Furthermore, the patient’s general health status must be precisely determined during anamnesis. Also, a general inspection regarding the nutritional status and comorbidities (diabetes mellitus, hypertension, neuropathy, systemic infections, vascular diseases, etc.) must be performed [16]. 

To date, six types of chronic wounds have been described: burns, diabetic ulcers, malignant ulcers, venous ulcers, pressure ulcers, and pyoderma gangrenosum, all of which are mainly characterized by specific clinical parameters, such as: granulation, slough, and necrotic tissue, with each type corresponding to a specific percentage of each type of tissue [27,28]. Out of them, diabetic foot ulcers (DFUs), venous leg ulcers (VLUs), and pressure ulcers (PUs) can be classified according to specific validated systems; namely, the Perfusion, Extent, Depth, Infection, and Sensation (PEDIS) classification system for DFUs, the CEAP classification (Comprehensive Classification System for Chronic Venous Disorders) for VLUs, and the National Pressure Injury Advisory Panel (NPIAP) pressure injury staging system for PUs [29,30,31,32].

Classification of chronic wounds in specific categories allows an etiologically based treatment, relying on systemic evaluation of underlying comorbidities, and can help predict outcomes [16]. Although biopsy of the wound tissue is considered the gold standard to measure the morphological changes of the wound tissue, other non-invasive and less time-consuming methods have replaced it. For example, laser Doppler imaging, indocyanine green video-angiography, spectral imaging, in vivo capillary microscopy, and many others [16,33]. Published reports highlight the diagnostic and prognostic value of these non-invasive imaging techniques both in preventing and monitoring potential complications and in tracking treatment progression and outcomes [33,34,35,36].

### 3.2. Standard Wound Management Principles

**Figure 3:**
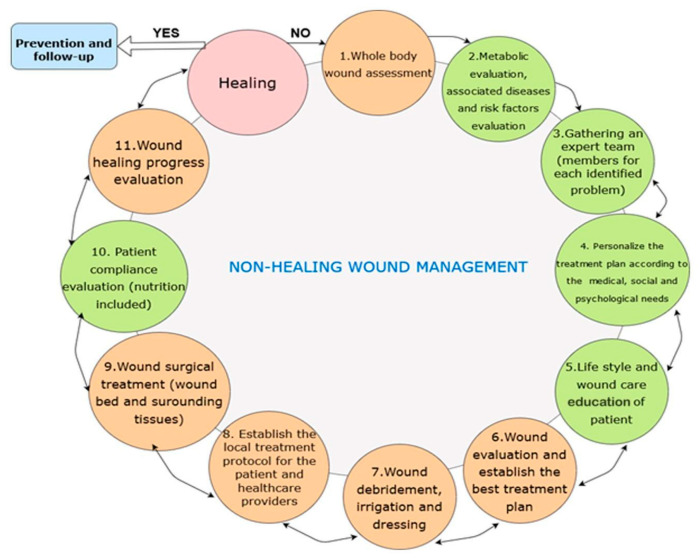
Chronic wound assessment algorithm. This system is based on a integrative perspective focused on the patient as a whole. Each step is as valuable as all others.

Management of chronic wounds is challenging, requiring a systematic and multidisciplinary approach to the patient, their comorbidities, and the wound itself. An essential part of the treatment algorithm lies in controlling metabolic, environmental and social factors that interfere with proper wound healing, including nutritional imbalances, vascular insufficiency, antimicrobial therapy, social habits such as smoking and alcohol abuse, and medications, as described in Figure 3. For example, in diabetic patients, tight control of their glycaemia, nutritional panel, and prevention and management of a potentially associated renal insufficiency can reduce adverse effects on the healing of DUs [16]. The first and primary goal in DU patients is to restore a palpable pulse in the affected distal lower limb. Revascularization can be achieved by endovascular or open bypass procedures or with hyperbaric oxygen therapy. VLUs are addressed with compression wrapping, with or without associated intermittent pneumatic compression, or surgery as a last resort [37,38]. Clinically non-infected ulcers should not be biopsied for culture, nor should the patient be treated with systemic antimicrobial therapy. However, chronic wounds complicated by the infection must be aggressively treated with debridement, surgical abscess drainage, and biopsy culture-guided antimicrobial therapy [39]. Even though debridement with scalpel and curette is considered the gold standard for wound cleaning, hydro-surgical or ultrasonic debridement may also be used, as well as maintenance debridement with collagenases or biodebridement with maggots [40,41,42].

For the past 15 years, an acronym has been used to summarize the main steps of a specific wound care approach [25,43]. It spells TIME and stands for Tissue assessment and management, Infection/Inflammation management, Moisture imbalance management, and Edge of the wound observation and management. To those principles, we can also add the apparent importance of offloading the chronic wound. Wound bed preparation, based on the TIME concept, represents a systematic assessment and treatment approach of chronic wounds. Therefore, each algorithm component needs to be personalized for each case and optimized to improve the chances of successful wound closure [24]. Wound bed debridement is the process of removal of the necrotic tissue in order to allow the inspection of underlying tissues. It must eliminate dead space, drain infections, and optimize the wound margins and wound bed so the reparation process may start or continue [24]. The optimal method of debridement must be patient-oriented and based on wound appearance, environmental factors, and medical staff available [23,24]. The leading cause of chronic wound failure is the imbalance of inflammatory cells, cytokines, growth factors, and/or proteases, such as matrix metallo-proteinases (MMPs) or the presence of biofilm [20,22,23,24]. High exudate levels may cause maceration and promote biofilm formation. In contrast, low levels may promote eschar formation and inhibit cellular proliferation [15,20,24]. In addition, the fluid secreted by chronic wounds has been shown to inhibit the migration and maturation of fibroblasts and increase the levels of reactive oxygen species (ROS), pro-inflammatory cytokines, and proteases [15,18,24].

Most patients will naturally respect the debridement measures, as the associated pain prevents them from applying pressure to the wounded area. In addition, specific devices can be used to help DFU patients, such as braces, removable or irremovable cast walkers, foot casts, and various foam dressings [16,44]. For VLU patients, Unna’s boot has three or four layers, and short stretch compression bandages [16,45]. Regarding topical wound therapies, there is no clear consensus on the most suitable approach, due to a lack of prospective data proving the efficacy of one therapy against the others. For this reason, clinicians have to select the most appropriate therapy based on personal experience and what seems best suited for each patient. Examples of topical agents range from povidone-iodine solutions to hypochlorous acid [16,46,47]. Similarly, there is no code for the most appropriate dressing, especially considering the myriad of commercially available products: standard cotton gauze, acrylics, hydro-fibers, honey alginates, micronized collagen, highly absorbent and moisture-retaining foam dressings, and many others [16,48]. As a general rule, the dressing should keep the wound moisturized enough without becoming a host for infection, protect it from external aggressions, and prevent pressure load [48]. 

Chronic wound care is mainly focused on covering, and in the past decade, numerous types have been developed in order to promote a moist environment that helps cells migrate and grow. Mankind has been dressing the wounds since forever, with the first wound covering having been described in 2000 B.C., containing mud, milk and different types of plants. The first indications about the management of chronic wounds dates back to 1550 B.C. [49]. Eastern European traditional medical knowledge mentions the use of various plants for skin treatment, such as *Brassica oleracea* L., *Matricaria chamomilla* L., *Arctium lappa* L., *Daucus carota* L., *Equisetum arvense* L., *Juglans regia* L., *Populous nigra* L., *Symphytum officinale* L., *Chelidonium majus* L., *Calendula officinalis* L., *Achillea millefolium* L., *Melilotus officinalis* L., *Allium cepa* L., *Quercus robur* L., and *Betula* spp. Studies on ethnobotanical therapy practices indicate that they are a cost-efficient alternative in therapy when used in a sterile manner [50]. 

The first step in wound management, following disinfection, should always be moistening. Moist wound healing prevents the evolution of the wound towards drying out, and has an important role in increasing the rates of reepithelization, while also improving the cosmetic look, and decreasing pain. Another important role of the dressing of the chronic wound is to absorb exudate. In this case, both calcium alginate and foam dressings are excellent at capturing and holding the fluid [51]. As wound management techniques keep improving, the more advanced dressings include an active step, which is the interaction between the wound and the biochemical environment [52]. 

The use of topical wound dressings did not demonstrate relevant effects in clinical studies on patients with chronic wounds. However, patients still use them so as to diminish the pain and improve the convenience of use (more extended periods between dressing changes) [49]. The standard medical care (the first-generation wound care products) in chronic wounds involves the assessment of both patient and wound, limited local pressure load, debridement of necrotic and/or infected tissue, treatment with antibiotics, and regular wound dressing changes [22,49,53]. The main target of the standard medical care development was to clean the wound and avoid site infection. Gauze dressings are made in various models: woven and non-woven fibers of cotton, rayon, or polyesters, to offer both absorbent qualities and protection against bacterial infection [24,53]. Some sterile gauze pads are designed to absorb exudates and fluids from an open wound using ultra-absorbent fibers embedded in the dressings. In the case of exudative wounds, more frequently changing dressings is necessary, because exposure of intact skin to moisture and friction causes erosion. Gauze dressings are the most common solution, but the downside is that they tend to drain the wound excessively, so the dressings get moistened and tend to become adherent to the wound. As a result of this adherence to the wound, the gauze change is painful and may destroy newly formed epithelium. The role of cotton bandages was initially for increased absorption of the exudate when placed as a secondary layer [54]. Modern versions include superabsorbent polymer (SAP) dressings which have been developed to cope with extra fluid that cannot be handled by standard dressings. However, these are currently used for high-compression bandages and short stretch bandages that provide sustained compression when treating venous ulcers [55].

The past three decades have focused on developing new types of wound dressings based on the evolution of biocompatible polymers that can provide a better moisture-thickness ratio and can ventilate the wound site with minimal risk of infection. Thus, non-adherent gauzes, silver impregnated meshes (Atrauman Ag, URGOCELL AG, Mepilex Ag, AQUACEL Ag+), hydrocolloid dressings (Hydrocoll, Duoderm), and the new generation of hydro-responsive wound dressing—HRWD (HydroClean, HydroTac)—were developed to boost the wound site healing process by regulating the wound bed medium [56]. The collagen dressing is also a good approach, as it improves healing with results similar to endogenous collagen. In this case, the collagen is obtained from cow, chicken, or pig, with small differences regarding the results [57]. 

Another available advanced dressing used lately is based on hyaluronic acid. This type is used mainly in venous leg ulcers. The last biomaterial introduced is amelogenin obtained from extracellular matrix proteins, with an important role in cell attachment. These advanced dressings are more patient-friendly and easy to use by a non-medical caretaker. Nevertheless, even with this progress in wound dressings, healing is not always possible. Therefore, further improving wound healing is a necessary step [58].

## 4. Novel Therapies

Advanced therapies available for wound management are indicated for chronic wounds in which the reparation process does not evolve after several weeks of standard care with adequate nutritional support. 

These therapies include negative pressure wound therapy (NPWT), which minimizes the wound volume and actively drains the excess fluids. At the same time, NPWT can improve blood circulation to the tissues where it is applied [59]. Also, topically applied platelet-derived products (PRP, PRF) as alloplastic cellular matrices with autologous growth factors and growth factors gels (Regranex, Endoret) are good options [60,61]. Additionally, we can include acellular extracellular matrices (MatriDerm, Theraskin, Integra, Matristem) for wound volume repair. Bioengineered ECM with cells included (Cuticell Epigraft, Apligraf, Dermagraft) for wound covering is another option [62]. Also, adipose tissue-derived stem cells are used to obtain as much regeneration in the wounded area as possible [63]. Other synergic treatments include hyperbaric or topical oxygen treatment to increase the wound’s oxygenation levels. Surgical treatment involving negative pressure wound therapy can be a reliable adjunct combined with surgical debridement. However, amputation may be unavoidable for locally advanced cases of wounds in which the abovementioned therapies cannot control the infection and severe tissue damage.

A modern approach to chronic wound care products is focused on developing autologous materials based on the patient’s cells to develop a biologic dressing able to regenerate the missing tissues and obtain faster healing. In this category, blood-derived products such as platelet-rich plasma (PRP), platelet-rich fibrin (PRF), adipose stem cell-based treatment, free flaps, grafts, and 3D-printed autologous tissues are the least expensive.

PRF is a fibrin matrix containing the platelet network mesh and growth factors that ensure a favorable volumetric evolution of tissue defects [61]. PRP is a liquid component containing growth factors obtained from plasma and platelets, giving a locally increased healing capacity [60]. Thus, the indications for using PRF are related to wounds with good nutritional intake but structural defects (extracellular matrix between the wound’s edges). In contrast, PRP is used for stimulating local angiogenesis and fibroblastic proliferation. If, in terms of plasma derivatives, attaining them is relatively simple (via blood centrifugation techniques), when it comes to tissue printing, there is a complex process that requires a controlled environment and many preliminary steps. First, pluripotent cells are collected either by enzymatic lysis of adipose tissue or from epithelial layers. After the tissue collection and the enzymatic separation of the cells is performed, it follows the incubation and multiplication of the obtained stem cells, only to be constructed as layers that mimic the physiological architecture with the help of a tissue 3D printer [64]. This process is costly, challenging to implement in surgical theatres, and very time-consuming.

The extraction of mesenchymal stem cells from adipose tissue (ADSCs—adipose-derived stem cells) uses one of the most explored types of stem cell therapy in the treatment of chronic wounds. Mesenchymal stem cells are isolated from a suitable tissue source, often bone marrow, adipose tissue, or umbilical cord tissue. They are then cultured and expanded in a controlled environment to obtain enough cells for therapeutic use. After this step, isolated MSCs undergo characterization to confirm their identity using cellular markers of MSCs. This involves assessing their cell surface markers and ensuring that they possess the potential to differentiate into various cell types, such as osteocytes, chondrocytes, and adipocytes. The expanded MSCs are prepared for application by ensuring their viability, purity, and proper handling. They may be cryopreserved for long-term storage if needed. For MSC application, the wound bed is prepared by cleaning and debriding necrotic tissue to create an optimal environment for cell integration and wound healing. The cells are applied directly to the wound site, often through injection, topical application, or incorporation into a scaffold or dressing. This enhances the regenerative environment and promotes tissue repair. The stem cells have the power to repair the injured tissue with their regeneration capability and by producing pro-regenerative cytokines [65]. Obtaining ADSCs is similar to the PRP process. A canula is used to harvest the adipose tissue and then stem cells are obtained by centrifugation. ADSCs improve wound healing and closure, and tissue ultrastructure, while promoting neovascularization. Isolated by liposuction or excision of adipose tissue, ADSCs are clinically effective in treating chronic wounds secondary to radiation damage, chronic fistulas, and ulcers, including venous leg ulcers [66]. This population of stem cells facilitates angiogenesis, stimulates the secretion of growth factors and cytokines, and allows the proliferation of dermal fibroblasts through direct contact with cells. Additionally, they stimulate the paracrine activation during the re-epithelialization phase of the healing process [67]. ADSCs allow the regeneration of subcutaneous, dermal, and epidermal tissues, especially when combined with skin grafts containing extracellular matrix. In treating chronic ulcers secondary to peripheral arterial disease, Marino and colleagues observed a reduction in surface area, depth, pain associated with the wound, and improved oxygen saturation in all patients treated with mesenchymal stem cells derived from adipose tissue [68].

In cases where the bone or the tendon are exposed, surgical treatment could be the best approach. Surgical treatment could include flaps and grafts. Skin grafts use either artificial skin (allografts), donor skin (xenografts), or the patient’s skin (autografts), with an important role in improving venous ulcer healing. The grafts supply matrix and cells in order to accelerate the wound healing. Nevertheless, grafts produce an occlusive cover, where the production of growth factors is stimulated and used as a biostimulator. The disadvantage of the method is that bad management of the patient could lead to the rejection of the graft [69]. Skin flaps are a pivotal medical concept for surgeons involved in the management of chronic wounds. Their ability to address the challenges posed by chronic wounds through improved blood supply, tissue coverage, structural support, and tailored interventions significantly contributes to successful wound healing, but for this the arterial supply in the area near the wound must be of good quality. We will not focus on surgical techniques and skin grafting, because the aim of the study is to find alternatives.

An innovation in the management of chronic wounds is the use of NPWT. The favorable effect is that the vacuum created by the negative pressure applied to the wound can modify the process of wound healing by stimulating granulation and angiogenesis, the reduction of edema, and matrix metalloproteinases. Overall, the NPWT ameliorates the blood flow and reduces the bacterial colonization within the chronic wounds [66]. 

The use of artificial intelligence (AI) for wound care has recently been gaining attention as a potential approach for improving patient outcomes. AI-assisted chronic wound care involves the use of a computer-based system to assess the wound status and determine an appropriate treatment plan. The system is able to assess wound size and shape, analyze the wound bed and surrounding tissue, identify the infection type and severity, and suggest optimal healing strategies. The system can also monitor the patient’s progress, alerting medical staff of any changes that require attention.

**Figure 4:**
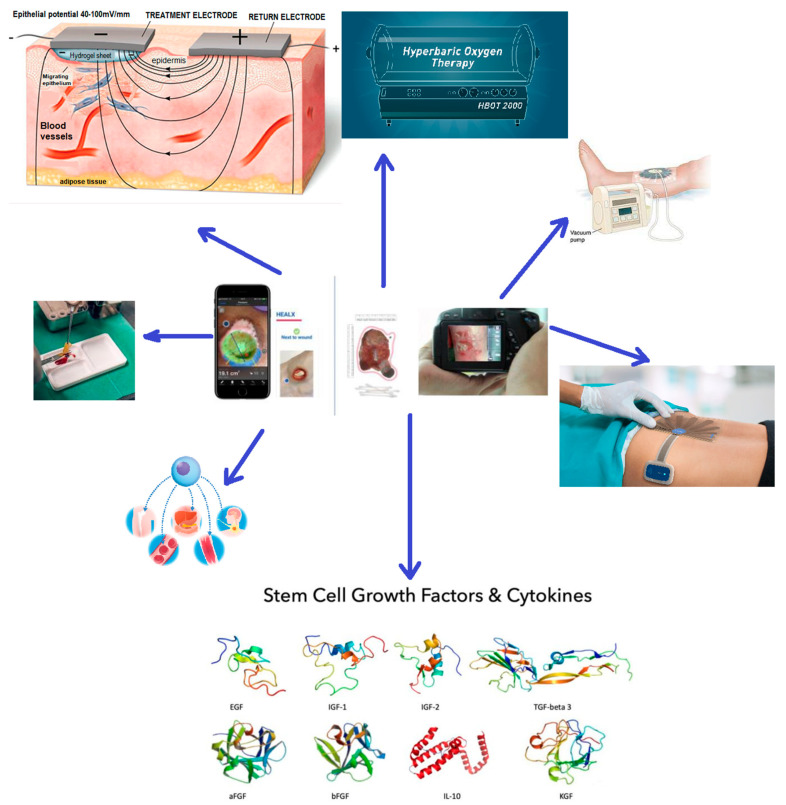
Future of chronic wound management. The AI software loaded on a smartphone ca predict and choose the most adequate therapy for the best outcome, by combining the latest available therapies.

Artificial intelligence based on large database comparison and analysis can now be used on computers, smartphones, or tablets. This new technique has enabled a rapid development and a modern way to enhance the diagnosis and efficacy of treatment in wound care in real time [70,71,72,73]. The apps are based on different types of algorithms that can analyze image data, medical parameters, or both, and can predict the outcome by comparison with the expanding database that they are linked with. A critical feature of the database and the AI that is using it, is that it is a proper data security system [74]. Ten years ago, we witnessed the revolution of artificial intelligence in the field of imaging like X-ray, CT, and MRI. Nowadays, the interest in the collaboration of medicine and computer science is steadily increasing, in order to obtain better patient outcomes for high-quality chronic wound care [75,76]. The apps are using many features as well as the size of the wound, the depth of it, localization, tissue characteristic, segmentation, and many others, added by manual entry. In this way, the physician benefits from greater documentation and a real-time monitorization of the patients’ evolution—see Figure 4. Chronic wounds that are currently using this kind of system are diabetic foot ulcers and pressure injuries. Footsnap, MOWA, SmarWoundCare, imitoWound, KroniKare, and Cares4wounds are only a few of the databases used nowadays for hard-to-heal wounds worldwide [77,78]. 

## 5. Conclusions

Chronic wounds are an important social and economic problem with a significant impact on patients’ quality of life and mortality. Currently, debridement, hydrocolloid dressings, and pressure-reducing surfaces are the main treatment methods, but new therapies such as biological with growth factors prove promising results in the future. 

The main treatment components for chronic wounds are wound cleaning, debridement, dressings, active infection control with antiseptics and/or antibiotic therapy, nutritional support, and vascular bed insurance. With the help of AI algorithms and machine learning, medical practitioners are now able to accurately diagnose and treat chronic wounds more quickly than ever before. AI-assisted wound care has been shown to reduce healing time and improve patient outcomes, while also decreasing the cost of chronic wound care.

Although additional studies are needed to demonstrate efficacy, modern therapies offer promising results and may, in the future, be adjuvant solutions to standard treatment for patients with chronic wounds. Of the biological materials, only skin grafts have shown efficacy in treating venous ulcers, improving the healing time associated with compression therapy. Both plasma derivatives (PRP, PRF) and mesenchymal stem cells derived from adipose tissue offer promising results in treating arterial ulcers, having an excellent safety and application profile in the surgical theatre.

Autografts or allografts can speed up the healing process of arterial ulcers. Adequate blood flow is needed, which can be achieved after revascularization, hyperbaric oxygen therapy, or other adjuvant treatment methods (such as PRP injections).

The AI system can also monitor patient progress, alerting medical staff of any changes that require attention. AI-assisted wound care has been demonstrated to reduce healing time, enable earlier discharge of patients, and significantly improve patient outcomes. Artificial intelligence (AI) has allowed medical practitioners to more accurately diagnose, monitor, and treat patients with chronic wounds, leading to improved patient outcomes and satisfaction. AI-assisted wound care also provides significant cost savings for hospitals, as the computer-based system can provide real-time data about a patient’s progress, thus enabling treatment election.

Based on the available evidence, the use of guidelines in chronic wound healing appears to be more effective than innovative local therapy in improving outcomes for chronic wounds. Guidelines provide a reliable and effective method for the treatment of chronic wounds, whereas the efficacy of innovative local therapy is inconclusive and needs more time to obtain significant statistical evidence. Furthermore, the implementation of guidelines can help to reduce long-term healthcare costs by encouraging control of risk factors, such as smoking and poor nutrition, that impede wound healing, and furthermore, by implementing them in the AI database. Therefore, the use of guidelines should be recommended as the primary treatment modality for managing chronic wounds and can be used in conjunction with innovative local therapies.

## Data Availability

No new data were created in this study.
